# Upon the Causes of the Greater Mortality of Male Children, and the Influences Operating to Change the Relative Proportions of the Sexes at Birth

**Published:** 1850-04

**Authors:** 


*Upon the causes of the greater mortality of male children, and the influence operating to change the relative proportion of the sexes at birth.* By G. Emerson, M. D.

Up to the fifteenth year, there is an excess of 15 per cent. in the number of boys over that of girls. This excess in the male mortality is commonly ascribed to the greater exposure, and rougher sports and amusements of the boys; an erroneous idea, the fallacy of which is shown in the fact that the majority of the deaths of the males takes place in early infancy, when no such exposure and danger consequent to said rough sports can possibly exist. The deaths of boys, too, from climbing, swimming, &c., equal those of the girls from scalding, domestic accidents, &c.

The particular diseases which give rise to death in the two sexes, are very different in their nature and characteristics. Thus, males are attacked with violent inflammation of the brain, accompanied with serous effusions, convulsions, &c.; inflammations of the stomach, lungs, and other important organs; while females suffer from hooping-cough, small-pox, measles, thrush, &c. In boys, the character of the disease is sthenic; in girls, asthenic. The diseases from which females suffer most are seated in the cutaneous and mucous tissues.

Of 100,000 deaths reported by the Registrar-general of England, 31,671 were under the fifth year; and of these, 15,006 were females, and 16,665 were males. Of the above, the number of deaths from inflammation of the brain were 2550 males, and 2081 females; of dropsy of the brain, 1481 males, 1151 females; small pox, 313 males, 240 females; hooping-cough, 1115 males, 1445 females; measles, 1048 males· 1028 females, etc,

From these and similar statistics, the inference follows that the disproportion in the deaths of the two sexes, during childhood, does not arise so much from exposure to external circumstances, as from differences in physical organisation.

From the fact of boys succumbing so easily and so rapidly to diseases of a sthenic type, and females to those of an asthenic character, we deduce the practical hint of combatting most energetically the inflammatory symptoms, of the one, as soon as manifest, and preventing too great exhaustion of the system when symptoms of depression begin to appear in the female infants.

The Dr. then spoke of the effects of weather upon infant mortality, and more particularly of the limitation of the effects of hot weather, to the period of lactation. For interesting facts relative to this subject, he referred to statistics lately published by himself in the Medical Journal of the Medical Sciences.

During the first year of infant life, the season of the greatest mortality is the three hot summer months. The number 250 representing the mortality for May, we would have 836 as tht for July. After the second year the deaths are more equally distributed throughout the months; the number seeming even less in the hot than in the temperate and cold seasons. The heat, which at an earlier period was inimical, would now appear to be friendly to infantile life.

Dr. E. next referred to the influence of certain agencies which changed the ordinary proportions of the sexes. The general preponderance of males over females at birth, is about 7½ per cent. In 1833 the singular phenomenon of a reverse proportion was evident. There was not only a deficiency of male births, but moreover, in the months of April and May of that year, a decided female excess. Upon further investigations, this female excess was found to be the product of conceptions occurring in the months of August and September of 1832. This, as is well known, was the period of the first invasion of epidemic Cholera. Looking abroad for corroboration of this singular fact, it was found to hold good also, in the proportion of births occurring nine months after the epidemic had appeared in Paris. From this and other investigations, he arrived at the conclusion, that this change in the relative proportion of the two sexes at birth, was owing to the depressing influence of cholera. He has further observed that a tendency to the above result is always produced by the operation of any class of depressing agents, while circumstances that tend to high physical development increase materially the male excess.

In France and Prussia, where the mass of the people labor much harder than in our own country, and are poorly clothed and fed, the excess of female births is slightly under 6 per cent.; in England, 5 per cent.; in Philadelphia, 7.5 per cent.; and in our western country as high as 10 per cent.

Investigations into the comparative proportions of the sexes born in city and country populations, manifest the existence of a greater male excess in rural districts. This, from the foregoing observations, was to be expected, since in cities, intemperance, foul and vitiated atmosphere, unwholesome diet, and other depressing agencies, operate much more strongly than in the country. Hence, the Dr. observed, this proportion of the births of the two sexes, may be considered as a sort of natural thermometer of the physical comfort and advantages enjoyed by a community.

The institution of polygamy may have originated in a scanty supply of food occurring at some former period in the community where such institution exists, and evincing its depressing tendency by a predominance of the female over the male population. Once established, it would foster itself.

The proportion of the two sexes being under such considerable control, it remains for the various legislative bodies throughout the civilized world to benefit and meliorate by their wise enactments the condition of the social cosmos.—*Philadelphia Medical Examiner.*

